# Trends for isolated amino acids and dipeptides: Conformation, divalent ion binding, and remarkable similarity of binding to calcium and lead

**DOI:** 10.1038/srep35772

**Published:** 2016-11-03

**Authors:** M. Ropo, V. Blum, C. Baldauf

**Affiliations:** 1 Fritz-Haber-Institut der Max-Planck-Gesellschaft, Faradayweg 4-6, D-14195 Berlin, Germany; 2 Department of Physics, Tampere University of Technology, Finland; 3 COMP, Department of Applied Physics, Aalto University, Finland; 4 Department of Mechanical Engineering and Materials Science, Duke University, Durham, NC, USA.

## Abstract

We derive structural and binding energy trends for twenty amino acids, their dipeptides, and their interactions with the divalent cations Ca^2+^, Ba^2+^, Sr^2+^, Cd^2+^, Pb^2+^, and Hg^2+^. The underlying data set consists of more than 45,000 first-principles predicted conformers with relative energies up to ~4 eV (~400 kJ/mol). We show that only very few distinct backbone structures of isolated amino acids and their dipeptides emerge as lowest-energy conformers. The isolated amino acids predominantly adopt structures that involve an acidic proton shared between the carboxy and amino function. Dipeptides adopt one of two intramolecular-hydrogen bonded conformations C_5_ or 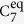. Upon complexation with a divalent cation, the accessible conformational space shrinks and intramolecular hydrogen bonding is prevented due to strong electrostatic interaction of backbone and side chain functional groups with cations. Clear correlations emerge from the binding energies of the six divalent ions with amino acids and dipeptides. Cd^2+^ and Hg^2+^ show the largest binding energies–a potential correlation with their known high acute toxicities. Ca^2+^ and Pb^2+^ reveal almost identical binding energies across the entire series of amino acids and dipeptides. This observation validates past indications that ion-mimicry of calcium and lead should play an important role in a toxicological context.

Proteins are the machinery of life. Their function is directly linked to their structure and dynamics. Natural proteins are polyamides that are composed predominantly of the twenty amino acids that are shown in Fig. 1. Their three-dimensional structures are shaped and their dynamics are influenced by several well-known conformational factors: (i) intrinsic structural propensities of the individual building blocks, (ii) intramolecular interactions such as hydrogen bonding, salt bridges, aromatic stacking, and van der Waals interactions, (iii) the surrounding medium, via bulk effects as well as by specific interactions. While many of the details of protein structure arise only when specific amino acids are combined in a chain, a first perimeter of the available conformation space is already set out at the level of individual amino acids^1^,^2^. This includes conformational preferences, e.g., through rigidity of bond lengths and angles, or through preferred backbone conformations defined by torsion angle patterns due to steric constraints. Furthermore, steric demands of side chain rotamers, electrostatics, protonation propensity, specific chemical interactions with side chains, and other local molecular properties are already present at the monomer level.

A particularly important example of specific interactions between proteins and their environment is that with cations. About 40% of all proteins are known to bind metals^3^,^4^,^5^. For example, Ca^2+^ is essential for living organisms due to its important role in a multitude of functions, from cell signaling to bone growth^6^. Calcium mediated functions can be disturbed by the presence of alternative divalent heavy metal cations^5^,^7^,^8^. In particular, lead is understood to “partially mimic the function of Ca^2+^”^9^, with a range of specific, documented long-term detrimental neurotoxic effects as a result. On the other hand, the sometimes very different chemical action of lead in a toxicological context compared to Ca^2+^ has also been pointed out^10^. It should be possible to establish the overall chemical similarity of two different ions such as Pb^2+^ and Ca^2+^ across a large series of potentially ligating biochemical groups based on atomistic simulations. This task is, however, fraught with difficulty even for simple descriptors such as binding energies. The reason is the large space of possible molecular conformations that must be assessed with uniform accuracy for both ions across a large series, even for comparatively small ligating molecules. Without knowing what are the relevant conformers to consider, structural trends based on total or free energies would remain qualitative and prone to accidental omissions of relevant conformers. Empirical potential energy surface descriptions could certainly cover the relevant spaces, but accurate ion-molecule interactions present significant difficulty in empirical atomistic modeling.

In this work, we categorize the intrinsic structural properties of twenty proteinogenic amino acids and dipeptides, as well as their interactions with the series of divalent cations Ca^2+^, Ba^2+^, Sr^2+^, Cd^2+^, Pb^2+^, and Hg^2+^, based on a recently published, exhaustive first-principles dataset of their possible conformational-energy minima^11^,^12^. The complete dataset covers 20 × 2 × 7 = 280 molecular systems (cf. Fig. 1): 20 proteinogenic amino acid side chains attached to 2 different backbone types, either free termini or capped (N-terminally acetylated and C-terminally amino-methylated), in 7 distinct complexation states, i.e., either the isolated system or in complex with one of the six cations Ca^2+^, Ba^2+^, Sr^2+^, Cd^2+^, Pb^2+^, or Hg^2+^. Different protonation states of the side chains (basic and acidic amino acids, see Fig. 1) or the backbone (neutral and zwitterionic) were considered where applicable. The total number of conformers covered is 45,730. This is close to the limit of what can be accomplished computationally for a conformational search of this extent and for the extensively benchmarked^11^,^13^,^14^,^15^,^16^,^17^,^18^,^19^ level of theory used to create the database (density-functional theory (DFT) based on a van-der Waals corrected generalized gradient functional (PBE + vdW)^20^,^21^,^22^, see Methods).

We here study the local, specific bonding contribution of the amino acids and dipeptides in conjunction with divalent cations, but otherwise in isolation. This environment does, of course, not resemble biological conditions, where cations like Ca^2+^ highly interact with their surrounding environment, e.g., water molecules forming hydration shells. However, for peptide-bound cations, such water-ion interactions do not interfere directly with the ion-peptide interactions. From a modeling perspective, including solvent effects either implicitly (by polarization effects)^23^,^24^,^25^,^26^ or by direct calculations of free-energy differences from molecular dynamics is in principle possible. For the breadth of the conformational study presented here, though, such an attempt would introduce the inevitable ambiguity of *which exact* solvent environment and/or which model to consider, as well as (for an explicit treatment) a sheer amount of free-energy calculations that is significantly beyond the scope of the present work. In the present paper, we therefore focus on the large and precisely definable total-energy contribution emerging from the specific ion-peptide bond.

Specifically, we show how a large-scale and systematic computational effort enables us to reveal conformational and binding energy trends that would not be readily apparent from isolated case studies based on different, potentially disparate levels of theory or experimental setups. A particularly striking example is the remarkable similarity of Ca^2+^ and Pb^2+^ interactions with a broad range of biologically relevant ligands, proven below and indicating that the related observations in toxicology^9^,^10^ emerge directly from the fundamental underlying potential energy surface.

## Results and Discussion

### Trend 1: Size of conformational space

Figure 2 summarizes the PBE + vdW conformational energy hierarchies and overall numbers of conformers considered in our study. For the amino acids and dipeptides without ions, the number of minima with the size (number of atoms) and flexibility (number of freely rotatable bonds) of the side chains of the building blocks. Consequently, we predict only a few conformers (from below ten to a few tens) for the small amino acids and dipeptides without a side chain (Gly), with a short side chain (Ala), or with a constrained side chain (Pro). In contrast, thousand(s) of conformers are predicted for the amino acids with long and flexible side chains, especially Arg and Lys. This number is not surprising, since the side chains alone give rise to many different conformations that are close in energy. Tabulations of these possible conformations, so-called rotamer libraries^27^, are sometimes used in protein modeling in order to predict, for a given backbone conformation, a set of probable side chain conformations. Current standard rotamer libraries are either (i) derived from protein crystal structures applying filtering and selection criteria^28^,^29^ or (ii) based on carefully curated sets of experimental protein structures or model peptides (GGXGG) that were subjected to molecular dynamics simulations^30^,^31^. From the latter, rotamers can be obtained that do not carry the bias of the crystal structures, yet they still rely on the empirical parametrization of the underlying force fields. Our data set^11^,^12^ offers an alternative, empiricism free, basis for the derivation of rotamer libraries.

If the amino acids are instead coordinated to the positive ion Ca^2+^, the overall space of conformational minima contracts significantly (1,694 and 4,103 conformers overall for the amino acids and dipeptides, respectively). Simultaneously, the relative energy range expands to up to about 4 eV or 400 kJ/mol (Fig. 2). Evidently, the cation places a strong electrostatic constraint on the positions of electronegative atoms and therefore reduces the accessible conformational space. This effect is especially pronounced for amino acids with a flexible side chain that interacts strongly with the cation due a lone pair, as exemplified by the difference between the unprotonated and protonated pairs Arg/ArgH and Lys/LysH. Here, the protonation results in a Coulomb repulsion between the positively charged cation and the positively charged end group of the amino acid side chain. As a results, the overall number of conformation changes from 484 to 135 for Arg/ArgH + Ca^2+^ and from 188 to 52 for Lys/LysH + Ca^2+^.

### Trend 2: Conformational preferences

For the isolated amino acids, the preferred conformation types are schematically summarized in Fig. 3A. Isolated amino acids (with the exception of Arg) are found to assume one of three basic backbone conformations (type I, type II, zwitterionic) as their lowest-energy structure. There is a close relation between type II and the zwitterionic state: only a minuscule shift of the shared proton from the carboxylic group to the amino function converts one type to the other. Most of the lowest energy conformers feature the shared acidic proton between the backbone carboxylic acid function and the backbone amino-N. Thus, this group of lowest-energy conformers is either type II or zwitterionic. In the case of Arg, a similar conformation is assumed by the zwitterionic backbone carboxyl group and the side chain guanidino function. We caution that, in several cases, the conformational energy differences between the basic backbone conformations are rather narrow. In these cases, changes to the level of theory could alter the detailed hierarchy observed^16^,^17^,^18^,^19^. In fact, the comparison to previous first principles studies of amino acids shows that different methods of calculation (level of theory and basis set) predict different preferred conformations^32^,^33^. In experimental studies of aliphatic amino acids, both states (type II/zwitterionic and type I) have been observed^34^,^35^, also highlighting the close energetic competition of the different states. Nevertheless, the emergence of just a few basic preferred backbone conformations from the vast overall conformation space studied is remarkable and, as a trend, robust based on the PBE+vdW level of theory applied here.

For the amino acids interacting with Ca^2+^ cations, zwitterionic and neutral/uncharged state of the backbone can be clearly distinguished. As illustrated in Fig. 3B, the cation can either bind to the lone pairs of the amino and carboxyl groups in the neutral/uncharged state (a.k.a. charge-solvated structure) or interact solely with the deprotonated and negatively charged carboxyl group in the zwitterionic state (a.k.a. salt-bridge structure)^36^. The zwitterionic backbone state is more stable than the uncharged backbone state for 13 of 20 amino acid-Ca^2+^ systems (see Fig. 3B). The cation-amino acid complexes of the aliphatic amino acids as well as of Gly, Pro, and Lys are predicted to be zwitterionic for all different cations in this study. Thr and Asn prefer the uncharged/neutral backbone state when interacting with the divalent cations covered by the present study.

**Figure 1:**
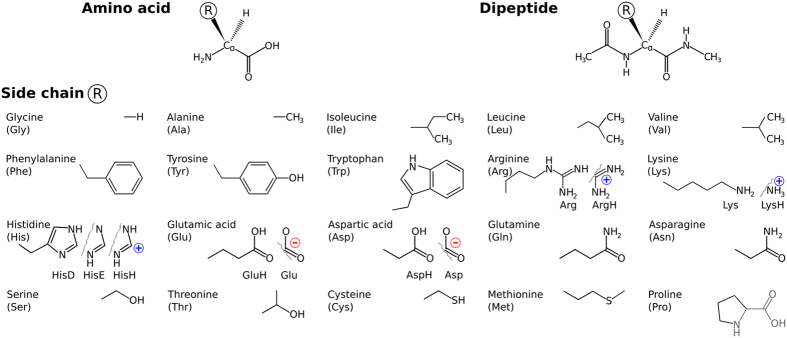
Molecular systems covered by this study. Top row: Basic chemical formulae of an amino acid and the corresponding dipeptide. Side chains are represented by **R**. Lower panel: The chemical structures for the 20 proteinogenic side chains **R** considered in this work. Where applicable, the alternative side chain protonation states considered in this work are shown as well.

**Figure 2:**
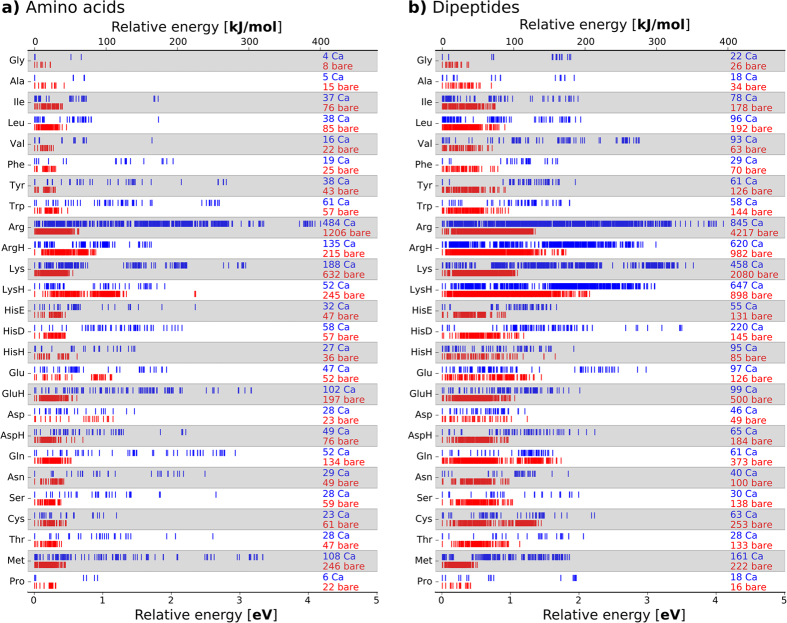
The conformational hierarchies for each amino acids (**a**) and the capped amino acids (dipeptides) are shown for the isolated (“bare”, red) and, alternatively, for the Ca^2+^ coordinated form (blue). The labels “Ca” and “bare” are accompanied by numbers that reflect the total number of conformers found for each system.

**Figure 3:**
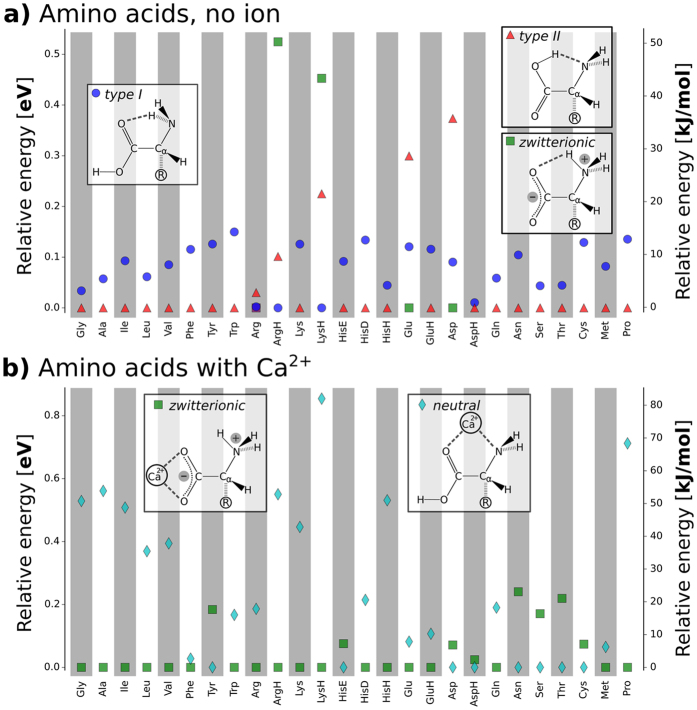
Preferred backbone conformations and protonation states for bare amino acids and for amino acids with Ca^2+^. **(a)** Schematic representations of the possible backbone H bonded structure types in amino acids together with a plot detailing the energy hierarchy of types I and II and the zwitterionic state for the isolated amino acids. **(b)** The two basic backbone-cation conformation types for amino acids with Ca^2+^ and a plot of their relative energies for each amino acid system studied. For clarity, only the lowest energy representatives of the respective structure types are shown. The energy of the respective global minimum is set to 0. The order of the amino acids on the *x* axis reflects chemical groups in the following sequence: aliphatic, aromatic, basic, acidic, amides, alcohol/thiol, other.

**Figure 4:**
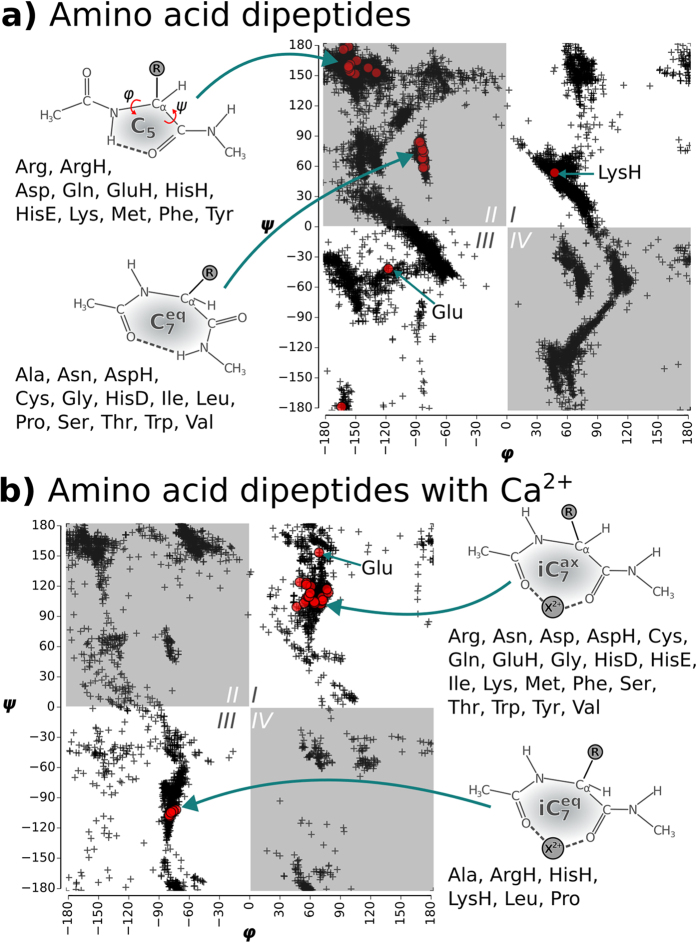
Ramachandran plots for the bare dipeptides **(a)** and for the dipeptides interacting with Ca^2+^ **(b)**. The *ϕ*/*ψ* tuples of the populated conformers of all dipeptides are shown by black crosses. The respective lowest energy conformers for each amino acid type are highlighted by red circles. Structural sketches illustrate the different dominant structure types of the global minima.

The amino-methylation and acetylation of the backbone functional groups of the amino acids leads to the so-called dipeptides, as schematically shown in Figs 1 and 4A. Since both termini resemble the local bonding environment of peptide bond, the dipeptide form is closer to the situation of building block embedded in a poly-peptide chain. In particular, end group effects such as a zwitterionic form cannot occur in the dipeptides. The backbone conformational space of the dipeptides can be represented by Ramachandran plots^37^ of the torsion angles *ϕ* and *ψ*. Figure 4A includes a graphical definition of both angles. The two dominant conformer types found for the dipeptides are referred to as 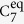 and C_5_. The nomenclature indicates the size of the hydrogen-bonded pseudocycle (5 or 7 members). The C_7_ pseudocycle can occur in two different conformations that are approximate mirror images. These images are, however, distinguished by the ***ax**ial* or ***eq**uatorial* orientation of the side chain ‘**R**’ relative to the plane of the hydrogen-bonded pseudocycle. The lowest energy conformers of each dipeptide are highlighted in the Ramachandran plot in Fig. 4A. The area occupied by C_5_ is located at the 180/−180 degree border between quadrants II and III. The area occupied by 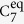 can be found roughly in the center of quadrant II. All dipeptides that preferably exhibit the C_5_ backbone conformation have bulky side chains. This is in accordance with the known propensity of bulky side chains to enforce the formation of *β* strand conformations. In contrast, the group in the center of quadrant II almost exclusively features members with comparatively small side chains, with the sole exception of Trp and its large indole side chain functional group that is also predicted to fall into the 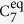 group.

The interaction with a Ca^2+^ cation has a major impact on the predicted conformations (see Fig. 4B). Structure types that are preferred without the cation, like C_5_ or 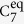, are hardly populated at all in the presence of Ca^2+^. Instead, there are now two new dominant areas in the Ramachandran plot in Fig. 4B that differ from the preferences exhibited by the isolated dipeptides. The global minima for most of the dipeptide-Ca^2+^ systems are located in quadrant I. The backbone oxygens of the acetyl moiety and the amino acid carbonyl group bind the cation. The corresponding structure is schematically shown in Fig. 4B. The cation is bound to the two backbone oxygens and thus closes an otherwise incomplete 7-membered pseudocycle (iC_7_). Similar structures were observed before by simulation and experiment for the interaction of sodium cations with small model peptides^14^. The side chain ‘**R**’ is oriented parallel to the axis (“axial”) of this pseudocycle and is hence referred to as 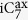. This orientation also allows for interactions between cation and respective side chain functional groups. For LysH, ArgH, HisH, Ala, and Leu, the predicted global minima are located in quadrant III. The corresponding backbone structure is again characterized by a 7-membered ring that is closed by the cation. For this group of dipeptides, the side chain is oriented equatorially, i.e. it is within the plane of the pseudocycle. Hence, the conformation is referred to as 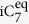. It is the approximate mirror image of 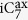. The preference for the equatorial side chain orientation is particularly strong for in case of the protonated sidechains of LysH, ArgH, HisH due to charge repulsion. Finally, Pro is conformationally restricted due to the heterocycle bridging the backbone.

### Trend 3: Ion binding energies

In addition to the structural effects of the cation, the binding energy of the individual amino acids to Ca^2+^ and to other divalent cations (Ba^2+^, Cd^2+^, Hg^2+^, Pb^2+^, or Sr^2+^) reveals distinct, remarkable trends across the series of amino acids and their dipeptidic form. Starting from the predicted conformers involving Ca^2+^, we created conformational energy hierarchies involving the other divalent cations by replacing Ca^2+^ with the respective alternative cation and re-optimizing the resulting structure. Importantly, we include all local structure minima found with Ca^2+^, not just the Ca^2+^-containing global minimum structure for each amino acid or dipeptide. In this way, we can faithfully compute the binding energies for all the alternative cations to the different proteinogenic amino acids and dipeptides, including the various protonations states. We define the binding energy from total energies of the lowest-energy conformations of the individual constituents as follows:


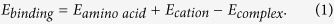


**Figure 5:**
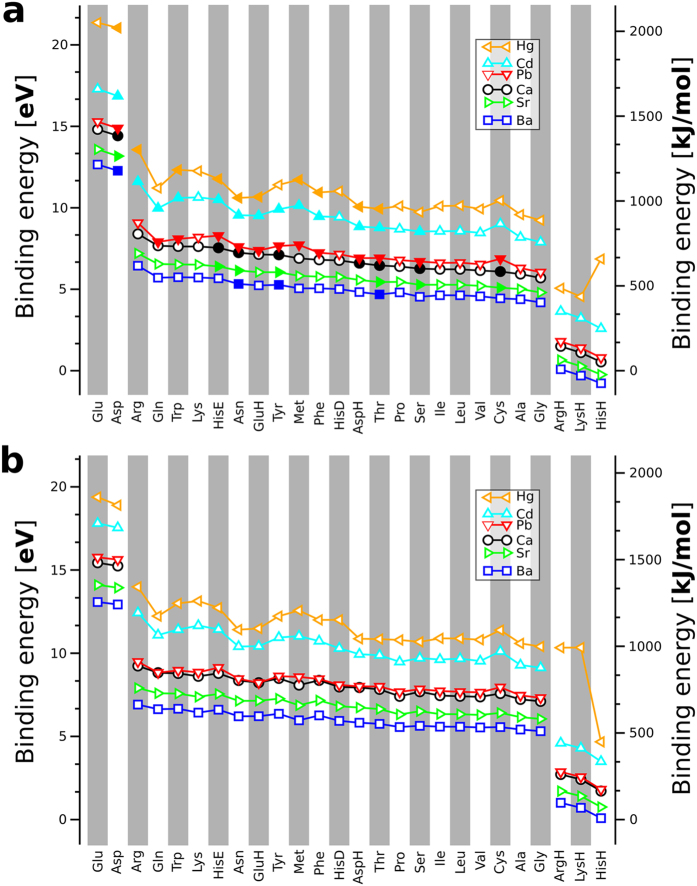
Binding affinity of the unprotected amino acids **(a)** and the dipeptides **(b)**. The building blocks are sorted according to their Ca^2+^ affinity with strongest to weakest affinity from left to right. Open symbols in **(a)** indicate the zwitterionic form and filled symbols the uncharged/neutral form as the respective global minimum. The amino acids and dipeptides (and their protomers where applicable) were sorted according to the binding energy to Ca^2+^ from the highest to the lowest values.

The order of the amino acids (or dipeptides) along the *x*-axis of the plots in Fig. 5 follows their affinity to the alkaline earth metals Ca^2+^, Ba^2+^, and Sr^2+^. The ranking for the amino acids binding Pb^2+^, Cd^2+^, and Hg^2+^ is similar but features slight deviations. Especially for the sulfur containing amino acids Met and Cys, the affinities to Pb^2+^, Cd^2+^, and Hg^2+^ increase more than for the other amino acids (dipeptides). The amino acids (dipeptides) can be roughly grouped according to their Ca^2+^ affinity (from strongest to weakest):


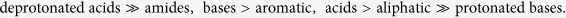


Electrostatic interactions are defining here, best illustrated by the high binding energies that result from the *attractive* interaction between the cation and the negatively charged deprotonated side chains of Glu and Asp and the low binding energies resulting from the *repulsive* interactions between cation and positively charged side chains in case of ArgH, LysH, and HisH.

Figure 5 also shows the increasing affinity of the cations to the amino acids (and dipeptides) following the order:


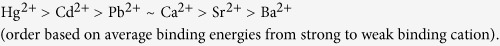


Importantly, the observed order of the binding energies of different cations holds uniformly across all amino acids and must therefore be intrinsic to the individual ions. We therefore exemplify an *a posteriori* explanation of this behavior by a comparison of the lowest-energy conformations for a few representative dipeptides (Glu, Arg, GluH, and Cys) as ligands to each of the six cations covered by this study.

Without loss of generality, we may discuss the interaction strength in terms of distinct contributions that are well established in chemistry: ionic (well defined if touching charged hard spheres are assumed), static polarizability, dispersion interactions, and, lastly, a contribution from covalent bonding that accounts for all remaining terms. We note at the outset that the computed differences in dispersion interactions between the six different ions and the peptides are much smaller than the trends shown in Fig. 5 and can therefore be neglected for the following discussion.

**Figure 6:**
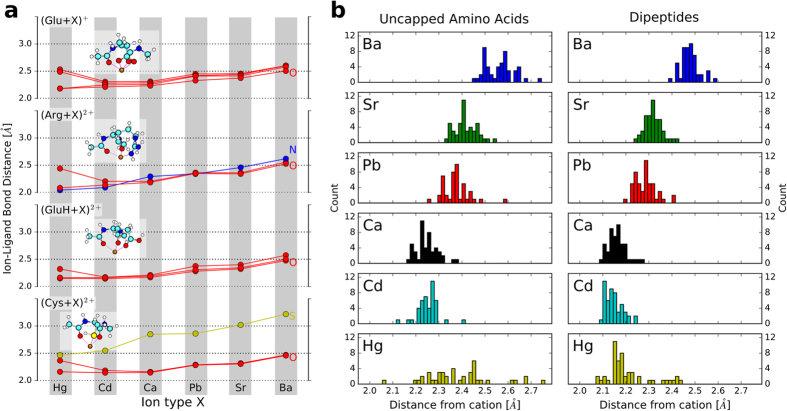
(**a**) Binding distances between the divalent cations and their nearest ligands in the lowest-energy conformations of cation-coordinated dipeptide forms of Glu, Arg, GluH, and Cys. The structure images in the insets show the Ca^2+^ coordinated forms and are structurally equivalent for the other cations as well. Different ligand atoms are distinguished by different-colored curves (red: O, blue: N, yellow: S), as noted in the figure. **(b)** Histograms of cation-O distances for lowest-energy conformers over all dipeptides or uncapped amino acids and the cations covered in the study.

In case of the alkaline earth metal cations (Ca^2+^, Sr^2+^, Ba^2+^), the binding strength trend is well represented already by the bond distances represented in Fig. 6a, which essentially reflect the increasing ionic radii from Ca^2+^ via Sr^2+^ to Ba^2+ ^38,39. For the pairs Ca^2+^/Pb^2+^ and Cd^2+^/Hg^2+^, however, ionic radii and ionic binding alone do not suffice to explain the observed trends. Covalent and/or polarization contributions must therefore account for the remaining differences.

For a more quantitative description in terms of our own data, we plot the ion-ligand binding distances for all six cations considered to Glu, Arg, GluH, and Cys in Fig. 6a. Pb^2+^ is in principle larger than Ca^2+^, consistent with our data. By purely ionic considerations, Pb^2+^ should therefore bind somewhat less strongly than Ca^2+^. However, Pb^2+^ features a relatively shallow filled *s* shell in Pb^2+^ that is absent in Ca^2+^. Thus, Pb^2+^ should be slightly more polarizable and may have a slightly larger covalent contribution to the binding strength, which results in the comparable binding energy trend for Pb^2+^ and Ca^2+^.

What sets Hg^2+^ and Cd^2+^ apart from Ca^2+^, Sr^2+^ and Ba^2+^ is a relatively shallow filled *d* shell for Hg^2+^/Cd^2+^. Ionic, static polarizability and/or residual covalent contributions should thus all lead to an overall stronger binding of Hg^2+^ and Cd^2+^ to the dipeptides than for the alkaline earth metals, as we observe. Between Hg^2+^ and Cd^2+^, however, Hg^2+^ is larger than Cd^2+^ in terms of tabulated ionic radii for the same coordination number^38^,^39^, and thus Cd^2+^ should bind more strongly for otherwise equivalent conformation. Yet, the opposite is the case in Fig. 5, i.e., Hg^2+^ binds significantly more strongly. The reason can be discerned from Fig. 6a. While Cd^2+^ retains approximately equal distances from its ligands, Hg^2+^ changes its local coordination shell to pull two of the ligands closer than the others, in fact closer than any of the ligands of Cd^2+^. This suggests a strongly covalent and/or static polarizability driven contribution to the Hg^2+^-dipeptide bond. The orbital-based explanation likely resides in the fact that the 5*d* shells of Hg^2+^ are shallower than the 4*d* shells of Cd^2+^. As a result, the frontier orbitals of Hg^2+^ are more flexible and have a larger incentive to participate in some form of residual bond. Overall, Fig. 6a reveals that the coordination chemistry of Hg^2+^ is in detail different from that of Cd^2+^, explaining the much stronger overall binding strength of Hg^2+^ to the amino acid and dipeptide ligands studied here.

The trend that we just discussed for a few selected amino acid dipeptides holds over the whole range of amino acids and dipeptides as is illustrated by plots in the Supplementary Information similar to the ones shown in Fig. 6a. Additionally, Fig. 6b shows histograms of cation-oxygen distances across the respective lowest-energy structures of all amino acids and dipeptides considered in this study. The histograms also reflect the trends of Fig. 6a, e.g. the increase of the median distances for Ca^2+^, Sr^2+^, and Ba^2+^ or the change of the one-peak distribution of Cd^2+^ to a multi-modal distribution for Hg^2+^ that indicates strongly covalent and/or static polarizability driven cation-O interactions existing alongside purely ionic interactions for Hg^2+^.

### Trend 4: Ion mimicry and toxicity

We next attempt to correlate toxicological effects of the different ions with their respective interactions across the series of amino acids and dipeptides. For acute (short-term) toxicity, we consider median lethal doses (LD_50_)^40^ for the chloride salts (selected because of their generally high solubility) of the respective divalent cations. The LD_50_ is the dose of a toxin required to kill half of the individuals in a test population; it is thus a measure of acute, not long-term toxicity. An interesting trend emerges when comparing these LD_50_ values to the binding energy trends reported in Fig. 5 of the present study. Ca^2+^ might naturally serve as a reference point in this comparison. The chloride salt is moderately toxic with an LD_50_ of 2,301 mg/kg. Sr^2+^ exhibits relatively low binding energies to the amino acids. The LD_50_ of the respective chloride is also moderate with 1,253 mg/kg. Toxic effects of Strontium result from radioactive isotopes and not from the binding competition of Sr^2+^ with Ca^2+^. Pb^2+^ and Ca^2+^ show almost exactly the same binding energy trend. Their acute toxicities are similar and not high, with LD_50_ values of 1,947 mg/kg and 2,301 mg/kg, respectively, for PbCl_2_ and CaCl_2_. The divalent cations of mercury and cadmium show the highest binding energies in our comparison. Interestingly, the acute toxicities of CdCl_2_ and HgCl_2_ are also much higher than those of SrCl_2_, CaCl_2_ and PbCl_2_, with LD_50_ values of values of 107 and 47 mg/kg for Cd and Hg, respectively. Indeed, cadmium ions bind tightly to proteins and compete with Zn^2+^ and Ca^2+^ and especially also with sulfur bound Cu^+^ and with iron in iron-sulfur centers in proteins^41^. For the cations Hg^2+^, Cd^2+^, Pb^2+^, Ca^2+^, and Sr^2+^, we might thus infer a tentative correlation of acute toxicities with binding energy trends. A clear outlier, however, is Ba^2+^. Figure 5 shows it to be the cation in this study with the lowest affinity to the amino acids and dipeptides. In contrast, its known acute toxicity is rather high, with LD_50_ values ranging from 100 to 300 mg/kg. Indeed, the toxic species is the bare cation and not (as in the case of Hg or Cd) the protein bound cation. Due to its large size and weak binding energy for a divalent cation, Ba^2+^ may interfere with K^+^ channels instead and consequently has effects on, e.g., the function of muscle tissue^42^,^43^.

We finally return to the remarkable, almost exact quantitative agreement of the computed binding energies for Ca^2+^ and Pb^2+^, respectively, in Fig. 5. This trend is here revealed across an entire *series* of molecular ligands that are central to biochemistry. Consistent with low acute toxicities, their similarity supports directly the available evidence that Pb^2+^ can mimic Ca^2+^ 9. Individual amino side group functions will not distinguish these two ions. Additionally, tabulated qualitative descriptors such as their standard hydration enthalpies (Pb^2+^: −1479.9 kJ/mol, Ca^2+^:−1592.4 kJ/mol)^44^ and Shannon ionic radii (octahedral: Pb^2+^: 98 pm, Ca^2+^: 100 pm)^38^,^39^ are also close. Yet, Pb and Ca are certainly not chemically identical^10^, e.g., due to possible multivalency of Pb. Thus, Pb^2+^ may “fly under the radar” of typical factors distinguishing ions, helping facilitate its more subtle (neuro)toxic actions^9^ or other detrimental effects on protein folding, maturation, and interaction in some instances^45^.

## Summary

This study is a first step towards an unbiased understanding of peptide cation interactions from first principles. A large, first-principles data base of more than 45,000 conformers of 20 proteinogetic aminoacids, their dipeptides, and their coordination with the six divalent cations Ca^2+^, Ba^2+^, Sr^2+^, Cd^2+^, Hg^2+^, and Pb^2+^ 11,12 allows us to identify trends across an entire series of biochemically relevant functionalities:

- In the gas phase and at the PBE + vdW level of theory, the type II/zwitterionic form of the amino acid backbone dominates over type I.
- The dipeptides (acetylated and amino-methylated amino acids) assume only two distinct types of low-energy conformations, C_5_ and 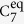.
- The conformational space of amino acids and dipeptides, as measured by the number of local minima found for each system, contracts upon coordination with divalent cations (with Ca^2+^ attachment, significantly fewer minima are found). Their conformational freedom is reduced by strong electrostatic interactions of backbone and side chain functionalities with the cation.
- The interaction of amino acids and Ca^2+^ occurs preferably via the deprotonated (negatively charged) backbone carbonyl functions. In the cases of the dipeptides, the Ca^2+^ cation preferentially interacts with the backbone carbonyl groups of the amino acid and the acetyl capping.
- The heavy-metal cations Hg^2+^ and Cd^2+^ bind more strongly to the amino acids and dipeptides than Ca^2+^.
- We can construct an *a posteriori* plausible correlation between the general binding energies of divalent cations to the amino acids and the acute toxicities of their chloride salts. The strong binding energies of Cd^2+^ and Hg^2+^ correlate with their much higher acute toxicities, while the weakest binder, Ba^2+^, is known to affect the function of another much more weakly binding cation, K^+^.
- At the PBE+vdW level of theory, Pb^2+^ shows binding energies to all studied amino acids and dipeptides that are virtually identical to those of Ca^2+^. Pb^2+^ thus has the ability to mimic much of the function of Ca^2+^, but not precisely, consistent with its interference with Ca^2+^ functions over longer time scales and/or in specific circumstances.

The insights generated in this work are inherently enabled by having access to an essentially exhaustive conformational energy data set for a wide range of amino acids, their dipeptides, and their various cation bound forms. The focus of our study is to find general trends across a large portion of chemical space. This of course comes at the expense of investigating more subtle effects, e.g. effects of electronic correlation energy treatments at higher levels and the effects of finite temperature, and entropy are not reflected but could be included in future refinements of the existing database. By considering only single amino acids/dipeptides we consider steric effects only to a limited extend. Next steps could include the reconstruction of protein cation binding sites in a similar fashion a outlined in the review articles by Dudev and Lim and in references cited therein^46^,^47^,^48^. In addition and as noted in the introduction, the environment or solvent medium surrounding cations bound to peptides is, of course, very important. For a quantitative understanding of specific environmental conditions, we believe that dedicated simulations incorporating effective continuum models^23^,^24^,^25^,^26^ or simulations of free energy differences by explicit molecular dynamics constitute excellent follow-up opportunities to our study.

Nonetheless, the emergence of unambiguous overall trends from an entirely non-empirical first-principles treatment (not relying on any specific “chemical intuition” at the outset) in our study is encouraging, showing the relevance of trends at the Born-Oppenheimer potential energy surface as a basic quantity and highlighting the power of systematically scanning large segments of chemical space with high computational accuracy to underpin empirically suspected trends.

## Methods

The level of theory employed to assemble the conformer database^11^ is a high-accuracy^49^ numeric atom-centered basis set implementation^20^,^50^ of density-functional theory (DFT) in the Perdew-Burke-Ernzerhof (PBE)^21^ generalized-gradient approximation, combined with the pairwise van der Waals correction by Tkatchenko and Scheffler (PBE + vdW)^22^. The reliability of PBE + vdW for the peptide structure problem has been established by (i) comparisons to CCSD(T) benchmark investigations for oligo-alanine systems^13^ and alanine dipeptides with Li^+ ^14, (ii) comparison to carefully performed basis-set extrapolated MP2 calculations^11^, and comparisons to experimental spectroscopic benchmarks^15^. Importantly, the actual *conformations* identified by PBE+vdW remain meaningful even in cases where, for larger oligopeptides, more expensive higher levels of theory are needed to reproduce small energetic differences to the exact conformational energy *hierarchy* found in experiment^16^,^17^,^18^,^19^.

As described in detail in ref. 11, for each of the amino acids and dipeptides with and without Ca^2+^, a global basin-hopping search with Tinker^51^,^52^ was performed using the OPLS-AA force field^53^ to pre-screen for relevant conformations. This set of conformations was then relaxed at the PBE + vdW level. After this global search step, a local conformational refinement was performed by PBE + vdW based replica-exchange molecular dynamics (REMD)^54^,^55^,^56^ followed again by geometry relaxations with PBE + vdW. For a subset of seven dipeptides (Ala, Gly, Phe, Val, Ile, Trp, Leu) and by comparing to an independently performed genetic algorithm search at the same level of theory^57^, this search was shown to be essentially complete. Conformers for amino acids and dipeptides in complex with Ba^2+^, Sr^2+^, Cd^2+^, Pb^2+^, and Hg^2+^ were generated by substituting a different ion into the geometries of the Ca^2+^ complexes and locally optimizing the new geometry.

## Additional Information

**How to cite this article**: Ropo, M. *et al*. Trends for isolated amino acids and dipeptides: Conformation, divalent ion binding, and remarkable similarity of binding to calcium and lead. *Sci. Rep.* **6**, 35772; doi: 10.1038/srep35772 (2016).

**Publisher’s note**: Springer Nature remains neutral with regard to jurisdictional claims in published maps and institutional affiliations.

## Supplementary Material

Supplementary Information
