# Supplementary material for: Trends for isolated amino acids and dipeptides: Conformation, divalent ion binding, and remarkable similarity of binding to calcium and lead
Source: Sci Rep. 2016 Nov 3;6:35772. doi: 10.1038/srep35772 (PMC5093913; doi:10.1038/srep35772)
Supplement: Supplementary Information [file srep35772-s1.pdf]

Supporting Information

Trends for isolated amino acids and dipeptides: Conformation, divalent ion binding, and remarkable similarity of binding to calcium and lead

M. Ropo[1,2,3,*], V. Blum[1,4,*], and C. Baldauf[1,*]

[1]Fritz-Haber-Institut der Max-Planck-Gesellschaft, Faradayweg 4-6, D-14195 Berlin, Germany
[2]Department of Physics, Tampere University of Technology, Finland
[3]COMP, Department of Applied Physics, Aalto University, Finland
[4]Department of Mechanical Engineering and Materials Science, Duke University, Durham, NC, USA
[*]Corresponding authors: ropo@fhi-berlin.mpg.de, volker.blum@duke.edu, baldauf@fhi-berlin.mpg.de

Cation-ligand distances

Binding distances between the divalent cations and their nearest ligands in the lowest-energy conformations of 20 cation-coordinated proteinogenic dipeptides. Different ligand atoms are distinguished by different-colored curves (red: O, blue: N, orange: S), as noted in the following figures.

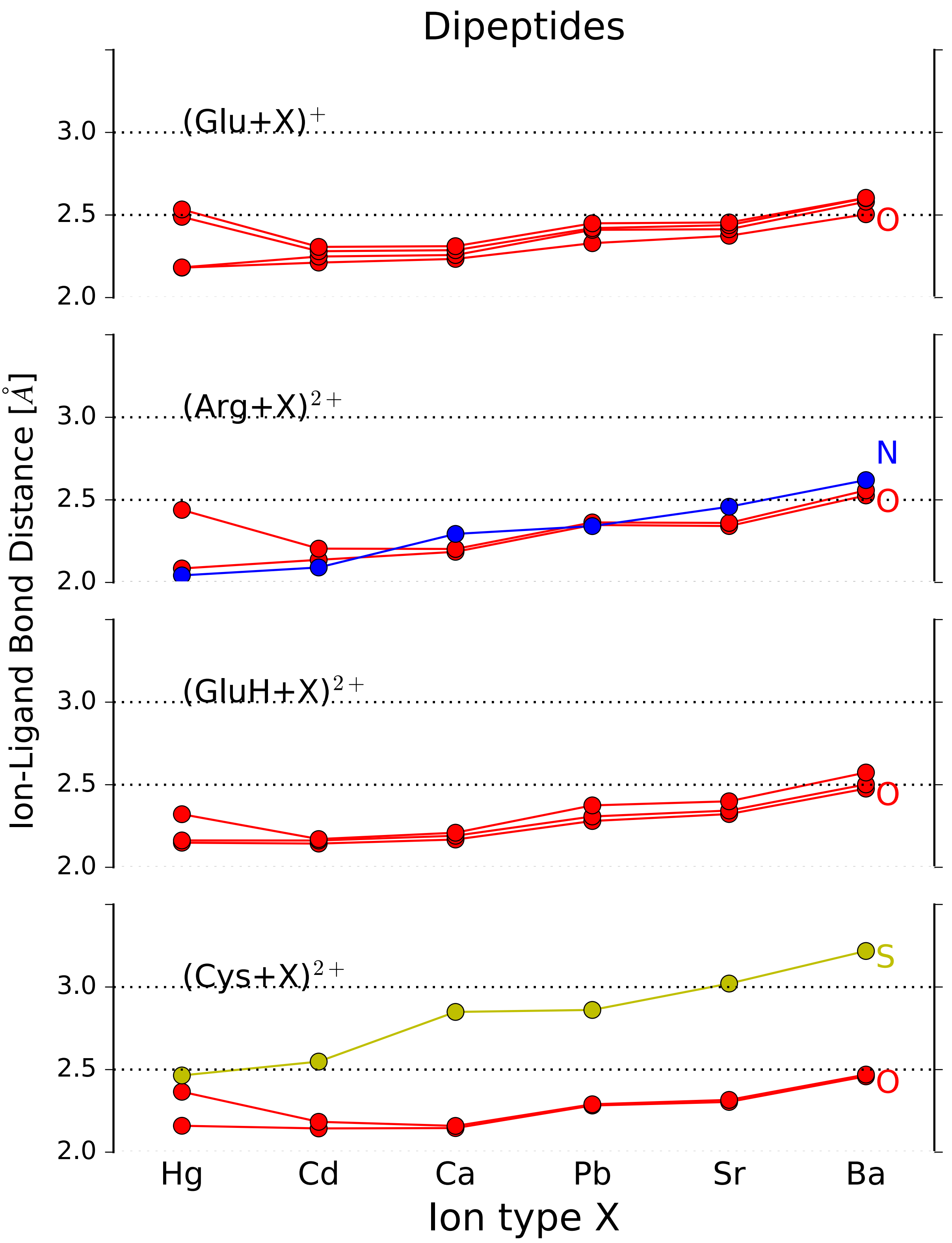
Dipeptides
(Glu+X)$^{+}$
(Arg+X)$^{2+}$
(GluH+X)$^{2+}$
(Cys+X)$^{2+}$
O
N
S
Ion-Ligand Bond Distance [Å]
2.0
2.5
3.0
Hg
Cd
Ca
Pb
Sr
Ba
Ion type X

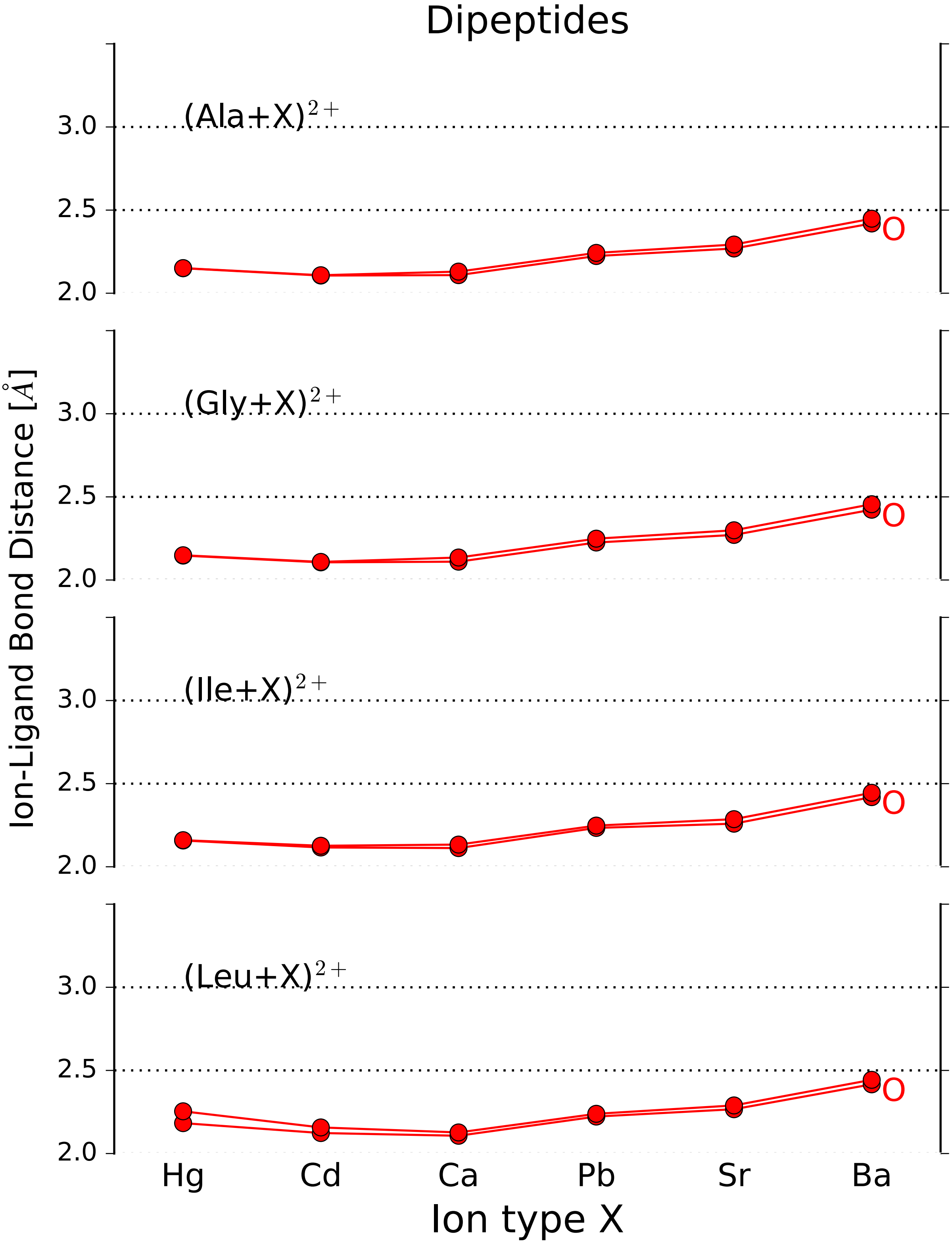
Dipeptides
(Ala+X)$^{2+}$
(Gly+X)$^{2+}$
(Ile+X)$^{2+}$
(Leu+X)$^{2+}$
O
3.0
2.5
2.0
Ion-Ligand Bond Distance [$\mathring{A}$]
Hg
Cd
Ca
Pb
Sr
Ba
Ion type X

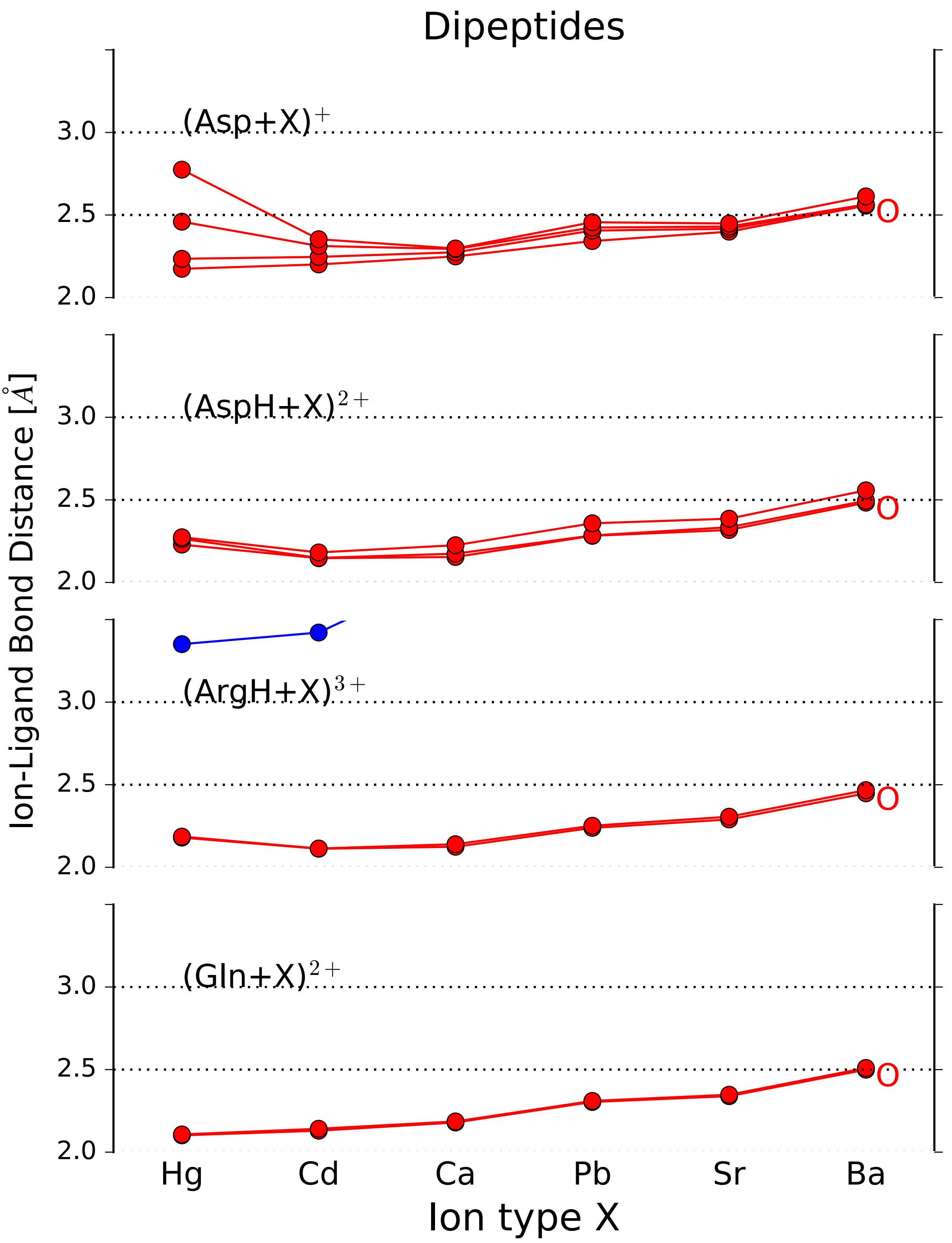

Dipeptides
(Asp+X)$^+$
(AspH+X)$^{2+}$
(ArgH+X)$^{3+}$
(Gln+X)$^{2+}$
Ion-Ligand Bond Distance [Å]
Ion type X
Hg
Cd
Ca
Pb
Sr
Ba
O
2.0
2.5
3.0

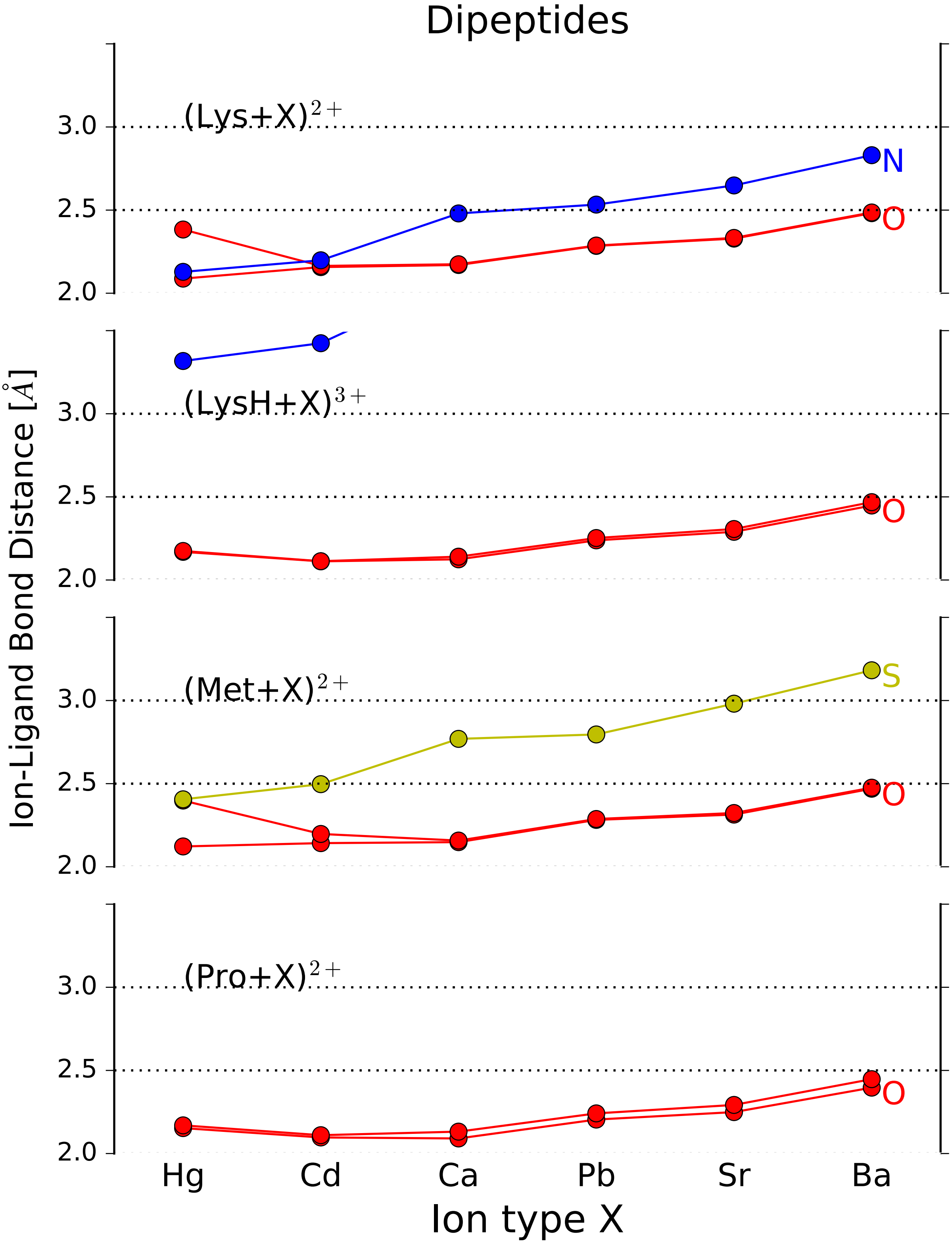

Dipeptides
(Lys+X)$^{2+}$
(LysH+X)$^{3+}$
(Met+X)$^{2+}$
(Pro+X)$^{2+}$
N
O
S
3.0
2.5
2.0
Hg
Cd
Ca
Pb
Sr
Ba
Ion-Ligand Bond Distance [Å]
Ion type X

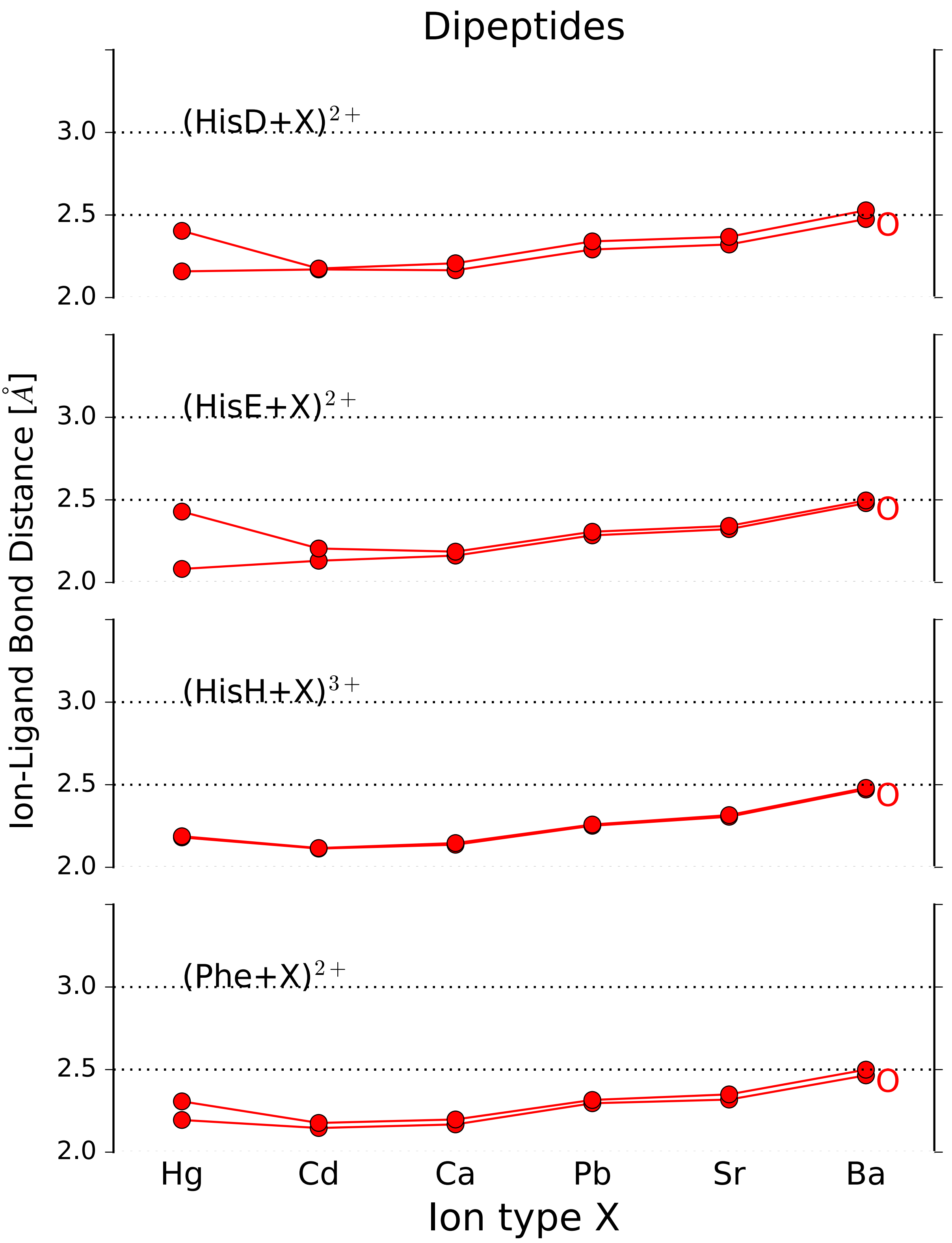
Dipeptides
$(HisD+X)^{2+}$
$(HisE+X)^{2+}$
$(HisH+X)^{3+}$
$(Phe+X)^{2+}$
O
3.0
2.5
2.0
Ion-Ligand Bond Distance [Å]
Hg
Cd
Ca
Pb
Sr
Ba
Ion type X

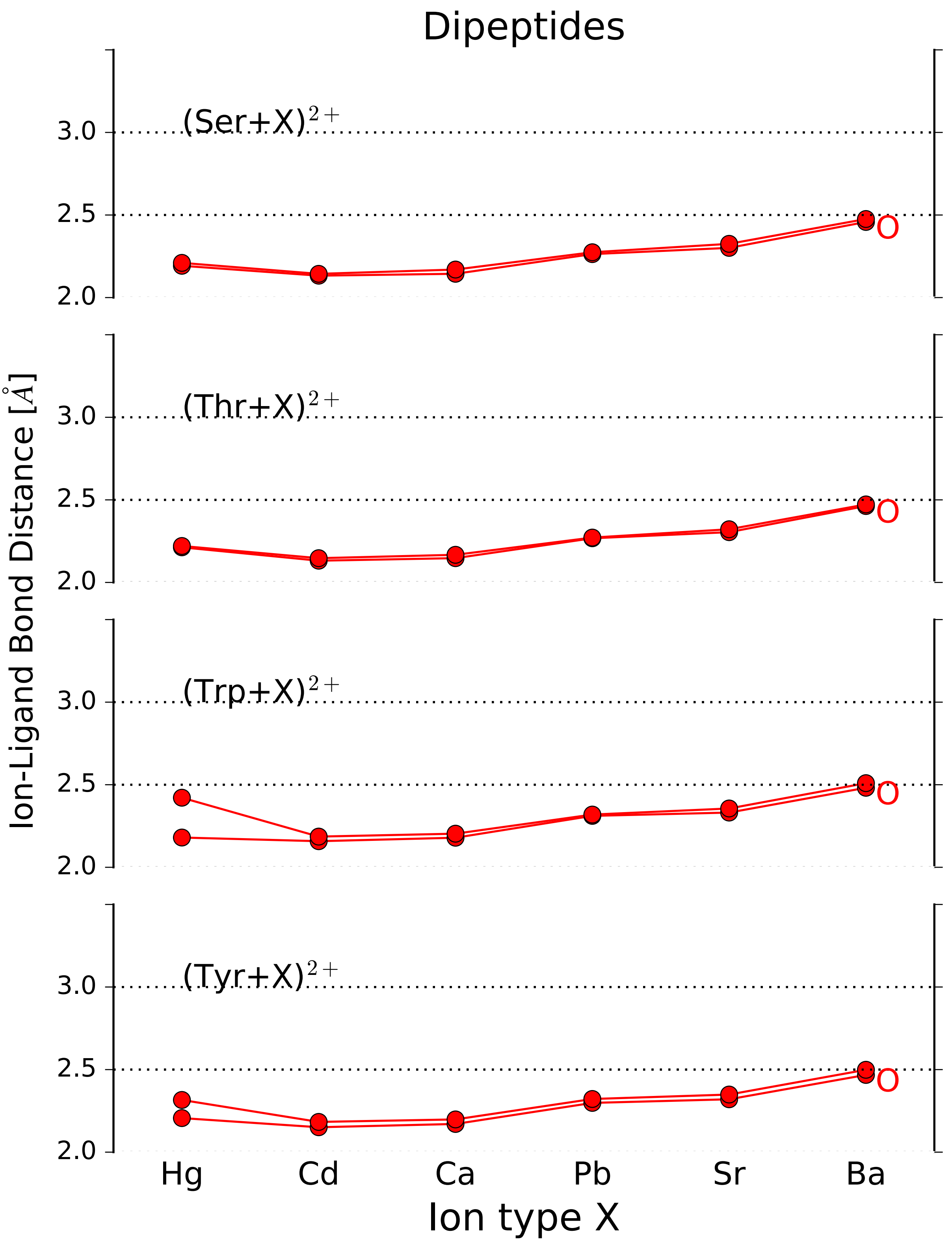

Dipeptides
(Ser+X)$^{2+}$
(Thr+X)$^{2+}$
(Trp+X)$^{2+}$
(Tyr+X)$^{2+}$
O
3.0
2.5
2.0
Ion-Ligand Bond Distance [$\AA$]
Hg
Cd
Ca
Pb
Sr
Ba
Ion type X
